# School development via the digital transition in nursing schools: Using empirical data to customize DigCompOrg for nursing education

**DOI:** 10.1186/s12909-026-09626-5

**Published:** 2026-06-15

**Authors:** Friederike Kalkmann, Simone Lienenbrink, Jonathan Behrens, Jannik Hoferichter, Gesa Borcherding, Christoph Bräutigam, Michaela Evans-Borchers, Johannes Laser, Tobias Theil, Manfred Hülsken-Giesler

**Affiliations:** 1 School of Human Sciences, Department of Nursing Science, University Osnabrück, Institute of Health Research and Education, Nelson-Mandela-Str. 13, Osnabrück, 49076 Germany; 2 Institute for Work and Technology, Westphalian University of Applied Sciences, Munscheidstraße 14, Gelsenkirchen, 45886 Germany

**Keywords:** DigCompOrg, Domain-specific, Nursing education, Digital transition, Digitalization

## Abstract

**Background:**

The digital transition increasingly poses challenges for educational organizations, as illustrated by the diverse approaches to digitalization processes adopted by German nursing schools. DigCompOrg, an European Union policy framework, addresses the complexity of digitalizing educational organizations and provides guidance on how to develop them accordingly.

**Methods:**

Five focus groups (*N* = 33) were conducted as part of a mixed-methods study. These focus groups aimed to address the question what nursing-specific characteristics influence the digitalization processes at nursing schools.

**Results:**

The level of digitalization at German nursing schools varies considerably. In addition to general conditions around nursing education, such as legal frameworks, institutional strategies, financial resources, and funding guidelines, the digital transition in nursing education is influenced by subject-specific factors. These subject-specific factors were found to be highly connected to the seven dimensions outlined in DigCompOrg. As a result, the specification of DigCompOrg was successfully defined in relation to nursing education at nursing schools. We found that facilitating the digital transition requires a shared professional understanding of nursing itself; cooperation between learning environments incorporating a third learning environment; critical reflection; and the pedagogical justification of technology use regarding body-related work in nursing. The specific characteristics of the profession must be considered alongside the existing dimensions of DigCompOrg in digitalization processes.

**Conclusion:**

A DigCompOrg framework specifically designed for nursing education can support the digital transition of nursing schools and can help schools focus their organizational development in the context of nursing education.

**Supplementary Information:**

The online version contains supplementary material available at 10.1186/s12909-026-09626-5.

## Introduction

Various national and international educational policy initiatives, increased public financial support and pandemic-related school closures during the COVID-19 pandemic have driven improvements in technical equipment at (nursing) schools [1–4]. Both in Germany [1, 5–10] and internationally [2, 3, 11], an increasing number of (nursing) schools are being equipped with digital technologies. When compared internationally, different institutional education systems characterize nursing education. Regardless of this, there is a common point of reference. In addition to the pandemic-related expansion of internet-based teaching and learning environments, including in nursing schools [3, 6, 7], technological innovations as well as national and international financial support are contributing to improved technical equipment in educational institutions [4, 12]. In Germany, the Digital Pact for Schools provided a total of €6.5 billion for the expansion of digital infrastructures in schools during a funding period from 2019 to 2024, leading to significant progress in their digital transition [13]. Internationally, adjustments have also been made through education policy initiatives, which have helped to reduce the “digital skills gap” in the vocational education and training (VET) sector [6].

Nevertheless, the state of digitalization in German nursing schools is mixed. While some schools have already implemented subject-specific technologies such as digitally supported skills labs, others still lack even basic digital infrastructure such as Wi-Fi, LMS, and servers [1, 7, 8, 14].

In addition to their digital infrastructure, nursing schools in Germany also vary in terms of instructors’ digital competence and the availability and use of subject-specific qualification programs [8]. Previous research has shown the relevance of digital skills among teaching staff for the successful implementation of digital teaching and learning technologies in (nursing) schools [1, 6].

Schools also vary in terms of the process design of digital transition; digital change often proceeds in an unstructured manner, and explicit digitalization plans are rarely established [7, 8]. Furthermore, the debate in Germany about suitable digitalization plans for the use of digital technologies in nursing education is still limited [14]. In contrast, the current discourse highlights the importance of systematic, concept-based approaches for the implementation of digital technologies in the context of educational practice and school organization [6, 7, 15]. Finally, there is widespread agreement that schools should consider digital transition to be a continuous process that not only addresses general technical development, but also teaching and learning processes, digital teaching skills and training formats, and organizational structures [16].

Despite the undisputed importance of theory-based frameworks as a basis for development and optimization processes in school development [17], there are currently no such models available designed for the context of digital transition in nursing schools in Germany. The key elements of European Union’s “Framework for Digitally Competent Educational Organizations” (DigCompOrg), shown in Table 1, provides guidance for the systematic planning of digital change in educational organizations, as well as for reflection and self-assessment of processes across school types, reflecting the complexity of digitalization processes in educational institutions [18]. The framework is based on a comprehensive international literature review, followed by expert discussions to validate and build consensus around it.

**Table 1:** DigCompOrg: Thematic elements and Sub-elements (Own Illustration based on Kampylis et al. 2015)

Leadership & Governance Practices	Integration of Digital-age Learning is part of the overall mission, vision and strategy
	Strategy for digitalage learning is supported by an implementation plan
	A Management and Governance Model is in place
Teaching and Learning Practices	Digital Competence is promoted, benchmarked and assessed
	A rethinking of roles and pedagogical approaches takes place
Professional Development^a^ In contrast to the other dimensions, the dimension „Professional Development” of the DigCompOrg framework does not have sub-elements	
Assessment practices	Assessment Formats are engaging and motivating
	Informal and Non-Formal Learning are recognized
	Learning Design is Informed by Analytics
Content and Curricula	Digital Content and OER are widely promoted and used
	Curricula are redesigned or reinterpreted to reflect the pedagogical possibilities afforded by digital technologies
Collaboration and Networking	Networking, sharing & collaboration is promoted
	A strategic approach is taken to communication
	Partnerships are developed
Infrastructure	Physical and Virtual Learning Spaces are designed for digital-age learning
	The digital infrastructure is planned and managed
Sector-specific element(s)	Sector-specific descriptor(s)

^a^In contrast to the other dimensions, the dimension „Professional Development” of the DigCompOrg framework does not have sub-elements

DigCompOrg comprises eight central dimensions: leadership and governance practices; teaching and learning practices; professional development; assessment practices; content and curricula; collaboration and networking; infrastructure, and sector-specific element(s).

These eight dimensions of the framework are not clearly separable, and are mutually interrelated. In addition, the framework includes an eighth dimension “sector specific element(s)”, which allows adaptation to particular educational contexts. However, this dimension has not been conceptually elaborated in detail and can be specified by users in a domain-specific manner [18, 19]. The framework is characterized in particular by the integration of the of leaders of educational organizations, teachers, and learners as well it offers the possibility of sector-specific extensions.

The framework was chosen in this study because it considers a range of different viewpoints (learners, teachers, school administrators), can be adapted regarding sector-specific elements and is based on a variety of previous models. Nevertheless, studies provide initial indications of the specific impact of digitalization processes on nursing schools such as structural conditions, the type of body-related work and subjectivizing action [20]. As well previous studies shown, that nursing schools have some way to go in terms of digital transition [7, 14]. One potential approach to support them could be the integration of domain-specific factors into one model. However, there is no comprehensive empirical data on the specific influencing factors.

Although DigCompOrg offers the possibility of this subject-specific differentiation, nursing-specific differentiations of DigCompOrg are currently not available either nationally or internationally, and no studies to date have reported any use of the framework in German nursing schools.

However, the DigCompOrg-associated self-assessment tool “SELFIE” (“Self-Reflection on Effective Learning by Fostering Innovation through Education Technology”) was widely used [18, 21, 22]. Hippe et al. [23] examined the use of the SELFIE tool by teachers and students at vocational education and training schools in an international study (*N* = 86,727). The study revealed that the tool was highly reliable in VET schools, and the construct validity of the questions was confirmed via psychometric methods. This finding indicates that DigCompOrg is also fundamentally suitable for vocational education contexts, although no findings are available for DigCompOrg regarding subject-specific differentiation or application in the context of (nursing) vocational education.

In the context of the current debate on the digital transition in nursing schools and the large variation in the digitalization of nursing schools in Germany [8, 9], this article presents a concept to further develop the framework specifically for the domain of nursing, using empirical findings from focus groups. The study presented here builds on previous studies. A standardized online survey with private and non-profit nursing schools in 2023 (*N* = 264) [8] and a full survey in 2024 (*N* = 359) at public, private and non-profit nursing schools [24] were conducted. A cluster analysis was carried out based on the data, showing a cluster typology of schools characterized by teachers’ participation in continuing education, digital infrastructure suitability, and the existence of a digitalization concept. Building on these results, qualitative interviews with administrators and teachers (*N* = 17) [9] were conducted in 2024 to identify conditions for successful digital transitions at nursing schools.

To further extend and deepen the findings from previous studies, we take relevant factors influencing the design of digital transitions and the subject-specific requirements of nursing education into account. The analysis of the empirical findings and the concept for the domain-specific further development of the framework are based on the research questions presented in Table 2. The target group for the domain-specific development of the framework are educational institutions in vocational nursing education. This article based on findings from the German nursing education system, which is primarily characterized by theoretical training at vocational schools in cooperation with practical training in nursing facilities [25].

**Table 2:** Research questions for the further development of DigCompOrg for nursing education contexts

Nursing-specific elements
Which domain-specific elements of the digitalization of nursing education institutions need to be taken into account?
Integration/location in the DigCompOrg framework
To what extent do domain-specific elements of nursing education interact with the existing dimensions of DigCompOrg? To what extent should the domain-specific elements of nursing education be considered separately? Can domain-specific elements of nursing education be located in one of the seven main dimensions?
The use of findings in school development
How should domain-specific elements be taken into account in designing and implementing the digital transition of nursing education institutions?

## Methods

### Research design

The empirical data underlying this study originate from the research project “Digitalization processes in vocational nursing education at nursing schools” (2022–2024) and were generated a part of a larger mixed-methods study. To answer the research question of this study, the data from five semi-structured focus groups (*N* = 33), were reviewed and evaluated to address the research questions. A qualitative approach was chosen to build on existing findings and identify new aspects of digital transition specific to nursing education, considering a variety of perspectives.

### Data collection

The focus group approach was chosen to understand how nursing schools approach digitalization processes and the factors that influence these approaches. The approach also aimed to explore shared perspectives within specific stakeholder groups through group discussion, recognizing that these perspectives emerge through interaction [26, 27].

The focus groups were conducted via videoconference in October–November 2024. Each focus group lasted approximately 90 min. All interviews were conducted in German. Quotations presented in this article were translated into English by the authors.

The focus group guidelines (the interview guide is provided in the Supplementary Information) were based on the main dimensions of DigCompOrg. The guide was used to discuss the implementation of digital transition processes at nursing schools in relation to each specific dimension, including experiences, challenges, and conditions for success. Furthermore, the group engaged in a discussion about what other aspects, beyond the framework, should be considered by nursing schools. The discussions were moderated using a semi-structured guide with open-ended and follow-up questions to encourage participation and ensure that all participants had the opportunity to contribute.

### Sample and recruiting

Five focus groups were conducted, each with participants from a single target group: teachers from nursing schools (*n* = 8); school administrators from nursing schools (*n* = 7); practice mentors in vocational nursing training (*n* = 5); experts from vocational training and nursing education (*n* = 7); administrative staff (*n* = 6).

School administrators and teachers were recruited using contact information voluntarily provided during an online survey conducted in summer 2024. Administrators were invited via email and asked to forward the invitation to teachers at their schools and to practice mentors in affiliated care institutions [8]. Participants in the teacher and administrator groups represented multiple federal states (including Bavaria, Baden-Württemberg, North Rhine-Westphalia, Lower Saxony and Hesse) and institutions run by public, private and non-profit providers. According to a previous study involving cluster analysis (*N* = 334), the level of digitalization of participants' schools varies. The cluster variables measured the availability of a digitalization concept, the evaluation of digital equipment and the proportion of teachers participating in further training on digitalization. The solution of cluster analysis had five clusters (very highly/highly/rather highly/rather low and low distinct digitalization profile), a silhouette coefficient of 0.6 and the rate from smallest (*N* = 50) to largest cluster (*N* = 100) was 2.0 [8, 24]. Thus, the sample included participants associated with schools at early stages of digital transition as well as those with advanced digital structures and activities.

Due to lower response rates, the practice mentors’ group was less diverse, including participants from Lower Saxony and North Rhine-Westphalia working in hospitals or outpatient care facilities.

Experts were identified by the research team and invited via email. The group included researchers in nursing and vocational education, nursing educators, and a representative of a professional association for educators. The administrative staff group included administrative employees of responsible organization for the nursing schools from Lower Saxony and North-Rhine-Westphalia, representing public, private and non-profit organiztions.

### Data analysis

All focus group discussions were audio recorded, transcribed verbatim by a professional transcription service, and checked for accuracy. The analysis proceeded in two stages according to Saldana [28]. First a model-oriented analysis was conducted in line with Philipp Mayring's [29] approach. Second, an inductive, exploratory re-analysis following Braun and Clarke [30] was conducted to identify themes not captured by the initial framework.

The initial analysis followed a qualitative content analysis approach based on Mayring [29], combining deductive and inductive strategies and supported by the software MAXQDA:

1. Application and refinement of a deductive coding scheme, including category definitions, coding rules, and an initial assessment of intercoder reliability, followed by revision and completion of coding.
2. Inductive summarization through paraphrasing, abstraction, reduction and grouping of coded segments.
3. Development of a structured results report including category definitions and illustrative examples.

Three units were defined: The coding unit consisted of meaningful segments within sentences, with sentences retained for context. The context unit encompassed consecutive segments that shared a common meaning. This allowed discussion sequences within the focus groups to be captured. The evaluation unit comprised all relevant segments aggregated during inductive analysis to enable abstraction and systematic structuring.

The main categories for the deductive analysis were derived from the research questions and the dimensions of the DigCompOrg framework [18]. To enhance trustworthiness, two researchers independently coded 20% of the materials and assessed intercoder reliability using percentage agreement and Cohen’s kappa. The evaluation of inter-coder reliability revealed a very low level of agreement. The coding scheme was subsequently revised through discussion to improve clarity and consistency. As this was attributed partly to the DigCompOrg framework, which does not assume strict delineation between dimensions [18], the remaining material was coded by a single researcher to ensure consistent interpretation of the framework dimensions. Finally, an additional researcher reviewed the results to ensure the intersubjective plausibility, and discrepancies were resolved through discussion.

Inductive summarization was applied to the coded segments. In line with the research objective, segments were paraphrased and abstracted to ensure that interpretations (a) were not tied to individual cases, (b) reflected shared experiences when no contrasting perspectives emerged, and (c) emphasized differences when experiences diverged. Less relevant content was removed, and similar elements were grouped. Increasing category stability across coding rounds indicated thematic saturation.

The initial framework-oriented analysis, which was closely aligned with the model, may have privileged certain aspects while obscuring others, thereby restricting the analytical scope. Therefore, a more open, data-driven second analytical cycle was conducted, following the principles of thematic analysis as outlined by Braun and Clarke [30]. It focused on identifying nursing-specific characteristics shaping digital transition in nursing education.

In the second cycle, an inductive and exploratory thematic coding approach was applied. First, the entire dataset was coded thematically. Codes were then grouped based on conceptual similarities and abstracted into higher-order categories. The developed categories were defined and delineated iteratively. The material was reviewed in relation to the developed categories to ensure consistent assignment of coded segments and to verify the fit between data and categories. The results were systematically documented in a structured format, including category definitions, paraphrased material, and anchor examples, to ensure transparency and traceability of the analytical process.

The findings were integrated by mapping identified characteristics onto existing dimensions and consolidating discipline-specific aspects, thereby extending the model. Reflexive team discussions accompanied the analysis process.

Previous study findings may have influenced interpretation, measures were taken to enhance trustworthiness. Initial coding was conducted by researchers not previously involved in the DibAP research project. Results from both analytical cycles were discussed within the research team. Nevertheless, further empirical grounding is needed to strengthen the transferability and robustness of the findings.

## Results

**Fig. 1:**
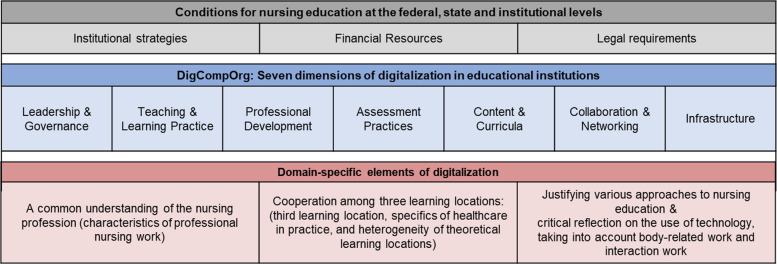
Domain-specific further development of DigCompOrg for the context of nursing education (Own Illustration)

Based on the empirical findings, we designed modifications to the DigCompOrg framework for the specific context of nursing education, as shown in Fig. 1. The figure illustrates the organizational and local/national conditions of the digitalization processes at nursing schools. The figure also incorporates the seven dimensions of DigCompOrg as a fundamental guide for school development processes during digital transition and also considers the newly identified domain-specific elements alongside the existing dimensions of DigCompOrg.

Notably, the political and institutional conditions for nursing education have a significant effect on the design processes involved in digital transition. The focus-group study also revealed that the seven main dimensions of DigCompOrg already address the requirements of digital transition in nursing education. The main dimensions were therefore included in the domain-specific framework. Our findings did not indicate any new dimensions that would need to be added. However, subject-specific elements that are particularly relevant for shaping digital transition in nursing schools were identified. These elements are closely related to the existing dimensions. As a result, we consider these subject-specific elements to be a cross-cutting area throughout the entire process of digital transition along the main dimensions of DigCompOrg.

The following sections provide a more detailed overview about results regarding the existing dimensions of the framework, the conditions for nursing schools in digital transition and sector-specific characteristics as a cross-cutting area.

### Main results regarding dimension 1–7 from DigCompOrg framework

The results regarding the *'Leadership & Governance Practice'* dimension emphasize the importance of systematic approaches, despite some schools not yet established explicit strategies. A heterogeneous openness to technology and an absence of unified professional understanding pose challenges. In *'Teaching and Learning Practices'*, the diversity of learners' digital competencies and domain-specific teacher profiling and reflection as learning facilitators in the nursing education process becomes evident as key areas of focus. Regarding *'Professional Development'*, the diversity of teachers' digital competencies and the resulting need for relevant offerings that address subject-specificity and account for this heterogeneity are also notable. The lack of available programs hinders competency development in this area. In the dimension of *'Assessment practices'*, the need for systematic justifications and reflection on profession-specific characteristics also is highlighted as a central aspect. Furthermore, legal regulations are identified as limiting the use of digital possibilities. Regarding the dimension *'Content and Curricula'*, limited integration of digital competencies in German nursing education curricula is evident. Additionally, the necessity of a shared professional understanding for curricular revision and the systematic integration of digital technologies is emphasized once again.

From a *'Collaboration and Networking'* perspective, the focus is on the diverse organizational structures of nursing schools in terms of their administration, as well as the associated coordination processes and cooperation. Collaboration in nursing schools is not only required in theory and practice, but also in the third learning environment, which must be systematically considered. In terms of *'Infrastructure'*, the heterogeneous digital infrastructure at schools, not widespread nursing specific technology and insufficient clear responsibilities and support structures become apparent. Furthermore, the high relevance of strategic considerations is evident once again here.

Overall, in addition to the conditions for nursing education at federal, state and institutional levels, and aspects already identified within the framework, cross-institutional collaboration, a shared understanding of the profession incorporating nursing-specific characteristics, and critical reflection on the appropriateness of technology use are particularly notable. The following section therefore presents the conditions for nursing schools as well as the three subject-specific aspects.

### Conditions for nursing education at the federal, state and institutional levels

In nursing schools in Germany, organizational possibilities are particularly dependent on the requirements specified by the school’s governing body. Nursing schools in Germany may be state-run or private (private/non-profit). These two different approaches to operation result in very different interests and strategies in some cases, e.g., with regard to integration into more complex digital infrastructures (e.g., through links to hospitals) or with regard to the training and continuing education of teaching staff. One participant in the focus groups noted:

We operate in different systems – in the education system at public schools, in the health system with its own logic, at private schools, which also have different teacher-training concepts or professionalization paths, and different continuing education and training; different ‘habits,’ I would call it (Focus Group “Experts”, Seg. 55).

These differences in the oversight of nursing schools can also be reflected in their digitalization strategies. While some schools develop their own school-specific concepts, others have to adhere to overarching digitalization concepts that may not only deal with educational institutions but also other institutions in the health and nursing care sector. These differences in oversight mentioned above have an impact on management practices and aspects of cooperation in the context of digital transition.

Depending on the type of institution (public or private), there was also clear heterogeneity in terms of the schools' financial resources and access to existing structures such as IT support within the institution. Regardless of public or private status of the institution, national funding programs with specific regulations (in Germany, for example, the DigitalPact for Schools) have a significant influence on the dynamics of school development in the digital transition.

In addition to the heterogeneous frameworks, resources, and legal status of schools, the legal requirements for nursing training must also be considered. The possibilities offered by digital assessment formats may conflict with legal requirements for examination formats:

I find these [digital] assessment formats very motivating and truly fantastic, but as long as we have the requirements in the Nursing Professions Act on how the exams are to be conducted, it is just a nice extra. Because I have to train the trainees in the formats, the exam formats […] specifically, the exam questions in the form in which they will be answered, or in the context of the oral exam. For all practical purposes, I think you can actually learn a lot from [Nursing] Anne [a nursing simulator], but that is a bit irrelevant. But if I have not learned how an exam is structured in three years of training, then I won’t be able to pass the written exam. I find that difficult. (Focus Group “Teachers”, Seg. 44)

### Cooperation among the three learning environments in nursing education

Learning environment cooperation encompasses the three learning environments in nursing training. Theoretical training takes place at nursing schools, which may be private or public, and thus have different relationships with the education system. The practical learning environment in nursing care facilities is characterized by working with vulnerable groups of people. In addition, there is a third learning environment in the form of Skills Labs, which play a particularly important role in improving the transfer of theory into practice by preparing students for practical work with vulnerable groups.

Synergy effects can also be specifically exploited through cooperation between schools to respond to the additional workload resulting from innovations such as the reform of nursing training and dynamic technological progress. In addition, the general culture of cooperation is weak, as one expert reported:

I do not know how it is in other vocational training areas. However, I feel that the culture of sharing is not emphasized much in nursing education. So, when I imagine the resources that are available, because everyone has already created a great lesson plan for every topic and tried it out and so on, and everyone collects these on their own desktop and they are not passed on, it feels like, I don’t know, it’s kind of weird. (Focus Group “Experts,” Seg. 84)

The focus groups also reported challenges in practical training due to a lack of coordination between nursing schools; for example, there are inconsistent assessment concepts and instruments for internships, as well as various regulations in schools regarding the use of artificial intelligence in training. This makes the work of practice mentor particularly difficult. Furthermore, administrators and teachers confirmed that cooperation could be helpful in developing ideas regarding the use of technology and nursing teaching concepts. The exchange of materials could save time, although this would require the development and provision of well-designed materials.

The results indicated, that the respondents saw particular potential for digital technologies in the context of cooperation and exchange between theoretical and practical training locations in the sense of theory–practice transfer. One example reported is the repeated creation of videos on practical nursing interventions as a medium to communicate assessment criteria of practical nursing interventions in a clear and transparent way. The creation and sharing of materials via digital learning platforms is therefore particularly useful in the context of staff shortages:

[I] think that this networking simply saves a lot of time and,..., not everyone has to reinvent the wheel. Because we have a nursing crisis and somehow, the time it takes to shoot these videos, to create these tasks, is all extra time that is added on top, which is then missing from nursing, so to speak. In addition, I would see that way too, so why does everyone have to reinvent it?" (Focus Group “Practice Mentor”, Seg. 478–481).

Furthermore, the respondents emphasized the relevance of the exchange of information and knowledge about digital technologies used in nursing (training) practice. This knowledge helps teachers to foster digital basic competencies among trainees, which support the use of technologies in practice. Exchange via digital platforms can also simplify communication about the absence and assessment of trainees. In this context, however, the respondents indicated their desire to see the establishment of uniform software programs to avoid technical compatibility problems. Nevertheless, teachers and practice mentors should continue to exchange ideas on training issues in face-to-face meetings as well as in digital networks.

In addition to theoretical training and the placement of trainees in real-world facilities, nursing training also includes practical instruction, which takes place at a third learning environment. The integration of the third learning environment into the training program is extremely important for the development of practical skills and the promotion of the transfer of theory into practice. In particular, the focus groups emphasized the relevance of “Skills Labs”; these enable trainees to simulate practical situations in various nursing settings and thus contribute to the integration of theory and practice:

So, everything is included [in the framework], but I’d still feel guilty if we did not mention the Skills Labs in this context, which actually represent a paradigm shift in teaching and combine educational and technical content, together with the equipment.... (Focus Group “Teachers”, Seg. 76)

In the discussion of the importance of digital technologies for cooperation, all three learning environments in nursing education should therefore be taken into account. According to the focus groups, the use of other digital possibilities, such as visualizing and experiencing typical work situations through virtual reality applications, is also increasingly conceivable with respect to the third learning environment. According to one administrative employee of responsible organization for the school, it is important to ensure that educators have the necessary knowledge and skills to actually use the Skills Labs, that a nursing education concept is in place, and that issues relating to the maintenance and service of the technologies and long-term financing have been clarified:

I once heard a something that I thought fit very well to the Skills Lab: you can have the most expensive Porsche, but it’s useless if you don’t have a driver’s license. In addition, that is actually very, very fitting, because if you have a really nice Skills Lab, which I’m all for, but no opportunities to work with it, then you switch to the topic of... “content and curriculum,” but also “refinancing,” that there are actually people who can take care of it, and that, as has already been mentioned, they do not have to invest time on the side or at home to deal with it. (Focus Group “Administrative Employee of responsible organization for the nursing school”, Seg. 85)

To successfully integrate new teaching and learning technologies into the classroom, it is therefore always necessary to train teachers in the relevant skills, make thoughtful curricular adjustments, take specific teaching and learning contexts into account, and clarify aspects of long-term care and maintenance of the equipment.

The aspects of cooperation in nursing education mentioned above are closely related to the DigCompOrg dimension “Collaboration & Networking.” The consideration of the third learning environment is also relevant in terms of the technical equipment of the Skills Lab in DigCompOrg dimension “Infrastructure”; the integration of nursing didactics into teaching fits in the DigCompOrg dimension “Teaching and Learning Practices”. In view of the frequently mentions of a lack of time resources, opportunities and spaces for cooperation among teachers should be established and supported by management through the DigCompOrg dimension “Leadership & Governance Practices.”

### A common understanding of the profession

Discussions about the importance of digital technologies for nursing education and training, as well as the development and provision of appropriate teaching concepts, require that the tasks and responsibilities of professional nursing are defined as clearly as possible. Only with this clear definition can a well-founded determination be made as to which subject-specific digital skills professional nurses need to have. According to the focus group participants, it is still unclear today which digital skills a trained nurse need:

...Perhaps there is also a lack of an overall picture of what digital skills we need for nursing professions when [nurses] are trained and working in medical facilities. I think there is a lack of strategies and programs in medical facilities as well. (Focus Group “Experts” Seg. 12)

In one focus group, an expert in nursing education reported that a common understanding of the nursing profession has not yet been sufficiently developed, even though this understanding is a prerequisite for a successful digital transition in nursing schools. Among those particular, nursing is still reduced to simple, task-oriented activities, and complex needs that would lend themselves to digital support are often hardly recognized:

And as I provocatively say, we do not need it for sponge baths, and we do not need it for round after round sponge baths either. In this respect, the question is, what is the task of nurses? That is why I always place so much emphasis on saying “professionals” – for me, that means nurses. In addition, we do not actually have any specific... Let us put it this way: when you get into a discussion with politicians who say, “However, nursing is always...”, they are very direct about what they want to achieve with digitalization. You cannot wash something digitally. (Focus Group “Experts”, Seg. 112)

However, a common understanding of professional nursing is indispensable, and this requires the involvement of all stakeholders in nursing education. In this context, the focus groups also discussed the understanding of digitalization as an interprofessional process:

I just believe that we still have the challenge of actually seeing the digitalization of healthcare as an interprofessional process so that patient care changes. In addition, at the moment, we are still in silos according to job titles and only think in silos (Focus Group “Experts”, Seg. 22)

The development of a common understanding of the profession and its central characteristics corresponds to the DigCompOrg dimension “Content & Curricula”. The common understanding of the profession is necessary to incorporate digital skills systematically into training. The DigCompOrg dimension “Collaboration & Networking” is also related to building a common understanding of the profession, as communication between the learning environments of theory and practice about the skills that nurses need is indispensable.

### Justifying and critical reflecting on the use of technology in nursing education

The focus group discussions also pointed out some critical perspectives on the increasing use of technology in nursing education contexts. For example, the largely unexpected push toward digitalization due to the contact restrictions imposed in response to the COVID-19 pandemic, as well as the largely parallel surge in funding to improve digital equipment at nursing schools as part of the DigitalPakt Schule (Digital Pact for Schools), often led to purchases that could not be sufficiently justified by nursing education concepts. Above all, a lack of time and human resources has led to pressure to “do something” and correspondingly purchase technology in these contexts. However, insufficient training opportunities for teachers on the use of these digital teaching and learning technologies with specific nursing education references are also significant in this context.

Critical reflection on the suitability of digital versus analog methods for the respective content and skills is key in a profession characterized by individuality, emotional labor, and body-related work:

Well, I always think, for example, about the special role of physicality in nursing education. Can you actually capture that through serious games, VR [virtual reality], or something like that? So, what special aspects need to be taken into account, so to speak, when I use different types of simulations, for example? (Focus Group “Experts”, Seg. 62)

In the context of skills development and digital innovation, the training of institutional digital skills must also be considered. This requires the identification of needs, which, according to statements made by a school administrator, is associated with challenges in terms of training in specific technologies used. Because various providers of practical training in nursing education exist in Germany, schools are confronted with the use of a wide range of digital systems that trainees might use. Taking all of these factors into account is difficult. In some cases, schools also lack access to specific documentation systems, such as those used in inpatient long-term care. The heterogeneous state of digitalization in practical training facilities also makes it difficult to determine needs. In some facilities, digital documentation is already firmly established; others still work with paper documentation. The results revealed that one strategy for addressing these challenges is to demonstrate and teach digital, practice-oriented technologies in theoretical training so that trainees are prepared for the use of digital technologies in professional practice. This requires knowledge of various systems; it is clear that this also ties in with the idea of cooperation between learning environment.

In sum, it is crucial to justify new nursing-education technology and critically reflect on its role, especially for body-related work. Doing so is related to the DigCompOrg dimensions “Teaching & Learning Practices” and “Content & Curricula.” The development of teachers’ skills in using technology for nursing teaching is linked to the DigCompOrg dimension “Professional Development.” Our findings furthermore show that communication between the learning environment of theory and practice is particularly relevant with respect to the content to be taught and the skills to be trained, so there is also a connection to the DigCompOrg dimension “Collaboration & Networking.”

## Discussion

### Domain-specific development of the framework

The findings of this study indicate ways to integrate nursing-specific aspects into the existing DigCompOrg framework and to consider the dimensions in interaction with each other. Our study did not find any additional dimensions for nursing education, but conditions for nursing schools and three subject-specific elements indeed overlapped with multiple existing dimensions. The conditions for nursing schools are characterized by laws, variety of requirements (public or private) and different funding opportunities. These influencing factors on a structural and organizational level were consistent with the results in a previous interview study [9].

The subject-specific elements include:

1. Cooperation between three learning environments:
  a A practical learning environment that reflects the specific characteristics of work in the healthcare sector (e.g., high vulnerability of clients, high demands on interprofessional cooperation, high demands on data protection and data security, etc.)
  b A theoretical learning environment that has to meet a wide variety of requirements due to heterogeneous (public or private) status (e.g., in terms of regulatory measures, financial frameworks, corporate strategies).
  c A Skills Lab learning environment (the “third learning environment”), which serves to improve the transfer of theory into practice and prepares students to work with vulnerable groups.
2. A common professional understanding of “nursing”: A specific understanding of the nursing profession as knowledge-based work, body-related work, interaction work, and work with limited standardization and complex arrangements [20].
3. The need to adequately justify and critically reflect on approaches to nursing education, taking into account interaction work and body-related work.

Successful cooperation between theoretical and practical learning environments is crucial in nursing education, as it facilitates the application of theory to practice. Our study highlights the need for an intense exchange of information regarding educational content, exam requirements, assessment criteria (in the context of the use of digital technologies for assessment), current technological developments in practice and digital technology standards. Despite the recognized benefits, such cooperation remains rare and digital technologies have primarily used for cross-institutional coordination of student placements and supervision so far [7]. Berger and Walden [31] classify cooperation among learning environments as ranging from no contact to continuous constructive collaboration, with the ultimate goal being sustained, in-depth exchange. Given the rapid pace of technological change, the continuous adaptation of digital teaching and learning tools is essential [31, 32].

The systematic integration of the third learning environment, such as a Skills Lab, is essential for practical skills training. Also digital mannequins, cameras, microphones and VR simulations are available, their systematic use is limited by a lack of didactic frameworks [33]. New learning settings that facilitate the transfer of knowledge from theory to practice should be incorporated into school development plans, taking into account organizational, teaching and personnel aspects [34]. Effective learning requires collaboration among teachers across schools [35]. Our findings show that, while cooperative communities for digitalization are desirable, they are not sufficiently implemented, and a culture of sharing between schools is largely absent.

A shared understanding of nursing practice is necessary to guide the development of digital skills. Professional nursing involves interactive, knowledge-based and complex work in uncertain situations [20]. Interviews revealed that digital technologies are often applied without didactic alignment to nursing-specific skills. General equipment often surpasses subject-specific resources, and readiness for subject-specific digital implementation remains limited until infrastructure is fully embedded pedagogically and didactically. Overall, cautious attitudes towards technology highlight the need to optimize infrastructure and develop nursing-specific didactic concepts.

When these technologies are used, they are not equally suitable for every teaching scenario, according to the statements made in the interviews, a critical reflection on the use of technology is necessary. In particular, the topic working in close physical contact with patients (a key characteristic of professional nursing) frequently arose in the debate on the subject-specific use of digital technologies. The characteristics of professional nursing work [20] must be taken into account when deciding on teaching and learning technologies and methods, such that the acquisition of skills is closely related to professional nursing practice. In this context, it should be noted that the DigCompOrg framework [18] takes an affirmative approach without critically questioning digitalization. However, particularly in the context of human-centered work, the question must be asked as to what limitations or disadvantages the use of technology entails. Despite the clearly recognizable positive aspects of technologies, negative effects should not be overlooked and should be considered in a normative and critical manner, especially in nursing science discourse [36]. Finally, although DigCompOrg focuses on the organizational level, the critical reflection should be supported by the institution.

According to the findings from qualitative interviews, promoting the establishment of critical reflection and the pedagogically grounded, meaningful use of digital teaching and learning technologies in nursing education may be achieved through the development of didactic concepts for technology use in nursing [9].

Furthermore, teachers must possess the relevant digital competencies specific to their subject area in order to implement subject-specific aspects. This includes developing a shared professional understanding on which to base the integration of digital competencies into teaching practice, as well as critically reflecting on and conceptually designing the use of technology in nursing education [1, 9, 14].

### Limitations

Our framework has been developed empirically, but has not yet been validated, so a further study for validation of the model is necessary before it can be used in a national context.

Furthermore, the study is limited to Germany due to the fact that the empirical data comes exclusively from German nursing schools and participants. The validity of the findings presented here for the broader European context still must be examined as well. Given the heterogeneity of nursing education structures, further research is essential to extend these findings to the European context: 68% percent of nursing education programs are offered through university, while the remaining 32% are offered at vocational schools [37], in contrast, nursing education in Germany is most being offered at schools. Nevertheless, some findings regarding care-specific needs probably could be transferable, it is necessary to examine the extent to which these assumptions are valid and whether, in a European context, further aspects of the digital transition specific to nursing education need to be taken into account.

Besides the lack of validation, there are limitations regarding the recruiting. The focus groups included practice mentors from only two federal states and did not cover the statutory long-term care sector. Teaching staff and practice mentors were recruited via school administrators, meaning that the opportunity to participate as a teacher depended on the school administrators. For practice mentors, participation depended on the communication between the school and the practice settings.

Further to this, the researchers from DibAP project conducting the focus groups may also have been influenced by prior findings from the study, including two online surveys and interviews. It was aimed to reduce the bias by involving other researchers in the analysis, however, a further empirical validation of the results is essential.

## Conclusions

Overall, this focus-group study provided the impetus for further developing the domain-specific DigCompOrg. Our modifications incorporate various specific aspects of the digitalization process (a common professional understanding of “nursing,” cooperation between learning environments with the integration of the third learning environment, and justifying and reflecting on teaching approaches and their relationship with the use of technology) as a cross-cutting area into the framework. This also entailed dealing with possible results and effects that are particularly relevant for nursing education (promotion of theory–practice transfer; teaching institutional-digital skills; teaching subject-specific skills by using digital learning technologies; and reconciling digital innovation with nursing as a fundamentally human-oriented profession).

Our modified framework is intended to support nursing schools in considering these special conditions and structures of nursing vocational training and in designing digitalization concepts and school development accordingly. The framework may provide guidance for incorporating elements such as learning environment cooperation, critical reflection on the use of technologies, and justifying various approaches to nursing education into the creation of digitalization concepts and implementation plans from the outset. This in turn could provide an impetus for exploiting the synergistic effects between practical and theoretical training locations and between schools. However, these potential contributions are still in the conceptual stage and require further empirical validation and refinement.

## Supplementary Information

Supplementary Material 1

## Data Availability

The datasets generated and/or analyzed during the current study are not yet publicly available, but are available from the Federal Institute for Vocational Education and Training (BIBB) on reasonable request.
