# Supplementary material for: School development via the digital transition in nursing schools: Using empirical data to customize DigCompOrg for nursing education
Source: BMC Med Educ. 2026 Jun 15;26:973. doi: 10.1186/s12909-026-09626-5 (PMC13267708; doi:10.1186/s12909-026-09626-5)
Supplement: Supplementary file 1 — Supplementary Material 1 [file 12909_2026_9626_MOESM1_ESM.docx]

**Focus group guidelines (reference framework: “DigCompOrg”)**

**1. Key questions on dimensions from the DigCompOrg framework model**

1.1 Business management

Which aspects of business management need to be taken into account, particularly at nursing schools, to ensure successful digitization processes?

1.2 Teaching and learning

Which aspects of teaching and learning need to be taken into account, particularly at nursing schools, to ensure successful digitization processes?

1.3 Professional development

Which aspects of personnel development need to be taken into account, particularly at nursing schools, to ensure successful digitization processes?

1.4 Assessment practices

Which aspects of assessment practices need to be taken into account, particularly at nursing schools, to ensure successful digitization processes?

1.5 Content and curriculum

Which aspects of content and curriculum need to be taken into account, particularly at nursing schools, to ensure successful digitization processes?

1.6 Collaboration and networking

Which aspects of collaboration and networking need to be taken into account, particularly at nursing schools, to ensure successful digitization processes?

Which aspects of collaboration and networking must be taken into account, particularly at nursing schools, to ensure successful digitization processes?

1.7 Infrastructure

Which aspects of infrastructure must be taken into account, particularly at nursing schools, to ensure successful digitization processes?

1.8 Sector-specific dimension

Which other nursing-specific aspects must be taken into account to ensure successful digitization processes at nursing schools?

1.9 Summary:

In which area of the dimensions just discussed do you see the greatest

need for adjustment with regard to nursing-specific aspects?

**2. Key questions for the development of self-assessment and external assessment tools for nursing schools**

The plan is to develop a self-assessment and external assessment tool for digitalization in nursing schools based on the refined model.

2.1 Key nursing-specific aspects

In your opinion, which nursing-specific aspects of digitalization should definitely be included in a self-assessment and external assessment tool to be developed?

What are the key success criteria for digitization in nursing schools from your point of view?

2.2 Application of the tool

Who should a self-assessment and external assessment tool be aimed at?

How would the tool need to be designed to be practical (discursive tool, highly operationalized assessment tool, or a combination)?

**3. Final key questions**

What would you need for an adapted DigCompOrg framework model to be helpful to you in practice? (Consider inhibiting and promoting factors, e.g., resources, workshops)

How can the subject-specific model be integrated into digitization processes at nursing schools?
